# A single-amino-acid substitution in the HA protein changes the replication and pathogenicity of the 2009 pandemic A (H1N1) influenza viruses *in vitro *and *in vivo*

**DOI:** 10.1186/1743-422X-7-325

**Published:** 2010-11-18

**Authors:** Lili Xu, Linlin Bao, Qi Lv, Wei Deng, Yila Ma, Fengdi Li, Lingjun Zhan, Hua Zhu, Chunmei Ma, Chuan Qin

**Affiliations:** 1 Institute of Laboratory Animal Sciences, Chinese Academy of Medical Sciences (CAMS) & Comparative Medicine Center, Peking Union Medical Collage (PUMC), Beijing, 100021, China

## Abstract

**Background:**

The novel pandemic A (H1N1) virus was first identified in Mexico in April 2009 and since then it spread world wide over a short period of time. Although the virus infection is generally associated with mild disease and a relatively low mortality, it is projected that mutations in specific regions of the viral genome, especially within the receptor binding domain of the hemagglutinin (HA) protein could result in more virulent virus stains, leading to a more severe pandemic.

**Results:**

Here, we found that a single amino acid substitution of Asp-to-Gly at position 222 in the HA protein of the A (H1N1) virus occurred after two passage propagation in the allantoic cavities of chicken embryonated eggs, and this single residue variation dramatically increased the viral replication ability in MDCK cells and pathogenicity in BALB/c mice.

**Conclusions:**

A substitution of Asp-to-Gly at position 222 in the HA protein was prone to occur under positive selection pressures, and this single amino acid mutation could dramatically increase the virus replication ability *in vitro *and pathogenicity *in vivo*. Our finding offers a better understanding of the transmission and evolution of the 2009 pandemic A (H1N1) virus and brings attention to further potentially severe influenza pandemic that may result from cross-host evolution of the influenza viruses.

## Background

On June 11, 2009, the World Health Organization raised the global pandemic alert level to phase 6, the pandemic phase, in response to the emergence and global spread of a novel A (H1N1) influenza virus, which emerged in Mexico in early 2009 [1]. The onset of the pandemic A (H1N1) influenza virus infection has been relatively mild, with clinical manifestations progressing from mild upper respiratory tract illness to severe pneumonia leading to acute respiratory distress syndrome [2]. Sporadic deaths happened in individuals with poor pre-existing immunity to influenza viruses. However, it is projected that mutations in the viral genome could result in more virulent viruses, leading to a more severe pandemic.

The virulence, pathogenicity, and host range of influenza viruses are determined by many factors, including virus-specific determinants encoded within the viral genome. The HA protein is especially important in that it main functions were receptor binding and membrane fusion. Glaser et al. reported that a single amino acid mutation at position 190 of the HA protein of the 1918 pandemic H1N1 influenza virus resulted in a preference for avian receptor (α2,3Gal sialic acid) to human receptor (α2,6Gal sialic acid) [3]. Also, the HA protein from the 1918 pandemic H1N1 switched from avian to human receptor specificity through mutations at two positions (Glu187Asp and Gly222Asp) [4]. In addition, the A/New York/1/18 strain of the 1918 pandemic possessed a Gly at position 222 and this mutation markedly affected receptor binding, reducing α2,6 preference and increasing α2,3 avidity [4]. Furthermore, a comparison of the HA sequences from the 2009 H1N1 viruses with that of the 1918 H1N1 viruses revealed a variation at residue 200, which is shown to be involved in receptor binding. It is proposed that this Pro-to-Ser substitution causes the relative avirulence of the 2009 H1N1 virus compared to the 1918 H1N1 virus [5]. More recently, Xu et al. reported that a single amino acid substitution of Gln226Arg in the HA of a H1N1 virus strain A/Solomon Island/3/06 resulted in the complete loss of binding to α2,6Gal sialic acid, and concomitant loss of the virus ability to replicate in the lower respiratory tract of ferrets [6]. All above studies demonstrated that polymorphism of the HA protein, especially within the receptor binding domain, play a critical role in the binding preference and pathogenicity of the H1N1 influenza virus.

In this study, two prototypic strains of the 2009 pandemic A (H1N1) influenza viruses were continuously passaged in chicken embryonated eggs. Afterwards, wild type and embryonated chicken eggs adaptive viruses were compared under *in vitro *and *in vivo *conditions. Virus replication was analyzed in MDCK cells, whereas pathogenicity was tested in BALB/c mice model. We hope that the results could further our understanding of the evolutionary event of the 2009 pandemic A (H1N1) viruses under environmental selective pressures when cross host, and the role of viral polymorphism of the HA protein in replication and pathogenicity of the 2009 pandemic A (H1N1) influenza virus.

## Materials and methods

### Viruses

A/California/04/2009 and A/California/07/2009 were the prototypic strains of the 2009 pandemic A (H1N1) influenza viruses which were collected in California, the United States in April 2009. Both of these two viruses were propagated in Madin-Darby canine kidney (MDCK) cells. The 50% tissue culture infectious dose (TCID_50_) was determined by serial titration of viruses in MDCK cells respectively, and the titers were calculated according to the Reed-Muench method [7]. Meanwhile, these two prototypic viruses were continuous passaged in allantoic cavities of 10-day-old chicken embryonated eggs, and mut-A/California/04/2009 and mut-A/California/07/2009 strains were the second passage viral stock propagated from the chicken embryonated eggs. All experiments involving the viruses were conducted under biosafety level 3 (BSL-3) conditions for both *in vitro *cells infection and *in vivo *animal challenge, in associated with guidelines of the World Health Organization http://www.who.int/csr/resourse/publications/swineflu/Laboratorybioriskmanagement.pdf.

### Genome sequencing and analysis

All eight gene segments of wild type and mutant A/California/04/2009 and A/California/07/2009 viruses were amplified by high fidelity PCR (KOD plus DNA polymerase, TOYOBO). The PCR products were purified and sequenced (Invitrogen, Shanghai). Sequences of all viruses were alignmented with CLUSTAL W (version 1.83) software.

### Cells infection

MDCK cells were maintained in Dulbecco's modified Eagle's medium (DMEM) (Invitrogen) supplemented with 10% FBS. 10^2 ^TCID_50 _viruses were added to cell monolayers in 35-mm dishes (Corning) separately. After a 60 min adsorption at 37°C, cells were fed with 3ml serum-free minimum essential medium containing tosylsulfonyl phenylalanyl chloromethyl ketone (TPCK)-treated trypsin (0.5 μg/ml) (Sigma) and antibiotics (Sigma). This was designated as 0 hour post infection. 100 μl viral supernatants were harvested at 12, 24, 36, 48, 56, 72, 96 hours post infection (h.p.i) and clarified from cell debris by centrifugation at 3,000 *g *for 10 min.

### Hemagglutination assay

After the MDCK cells were infected with each virus, the titers of viruses released into the cell supernatant were evaluated by hemagglutination assay with 1% turkey erythrocyte cells as an indicator of virus replication in the cells. Briefly, a 50 μl aliquot of virus sample was incubated with equal volume of 1% suspension of turkey erythrocyte cells for 30 min at room temperature. The hemagglutination titer was defined as the reciprocal of highest virus dilution that hemagglutinated turkey erythrocyte cells.

### Mice challenge

Female 5-week-old SPF level BALB/c mice (Institute of Laboratory Animal Sciences, Beijing) were used in the study. Mice were anesthetized and inoculated intranasally with 10^6 ^TCID_50 _of each virus in a volume of 50 μl, with 23 mice per group, in which 10 mice were monitored daily for signs of disease, weight loss, and mortality up to 14 days post inoculation (d.p.i). The remaining 13 mice in each group were euthanized at 5 d.p.i. and 10 lung tissues were collected separately for viral nucleotide material quantification and pathological investigation, whereas another 3 mice lung were collected for viral titer detection. The procedures were approved by the Institute of Animal Use and Care Committee of the Institute of Laboratory Animal Science, Peking Union Medical College (ILAS-PC-2010-002)

### Real-time PCR

Total RNA was isolated from the infected cell supernatants or homogenized mice lung tissues by using the RNeasy Mini Kit (Qiagen) according to the manufacturer's instructions. RNA was dissolved in 30 μl diethyl pyrocarbonate-treated water and stored at -80°C. First-strand cDNA was produced by using random primers with 8 μl RNA in a 20 μl reaction mixture containing 200 U Superscript III reverse transcriptase (Invitrogen). The Real-time quantitative PCR assays based on SYBR Green dye were performed on StepOne PCR system (ABI) with 2 μl cDNA in a 20 μl reaction mixture which also containing 10 μl of 2× SYBR Green PCR Master Mix (ABI), 1 μl each of 10 μM forward and reverse primers (SW-HA F786: 5'-AATAACATTAGAAGCAACTGG-3', SW-HA R920: 5'-AGGCTGGTGTTTATRGCACC-3'), and 6 μl nuclease-free water. Thermal cycling was done under the following conditions: 94°C for 3 min, followed by 35 cycles of 94°C for 30 s, 58°C for 30 s, and 72°C for 30 s. Fluorescence measurements were taken after each cycle.

### Pathological analysis

The mouse lungs were removed immediately following euthanasia, inflated, and fixed with 10% neutral buffered formalin overnight at 4°C. Subsequently, the formalin-preserved lung samples were embedded in paraffin and sectioned. Serial 4-μm sections were stained with Hematoxylin-Eosin and examined for pathological changes. The images were obtained on a OLYMPUS BX-50 light microscope with 40× magnification.

### Statistical analysis

Statistical analysis of the viral RNA load and titer were performed by SPSS 11.5 software and the DUNCAN and LSD methods were used for detection in one-way ANOVA means.

## Results

### Sequences analysis of the viral genomes

The full-length sequences of the viral genomes of wild type and egg-adaptive mutant A/California/04/2009 and A/California/07/2009 strains were obtained by high fidelity PCR and sequenced. Genomic polymorphism of the four viruses was showed in Table 1. In the HA protein, an Asp-to-Gly substitution at position 222 simultaneously occurred within mut-A/California/04/2009 and mut-A/California/07/2009 compared with wild type A/California/04/2009 and A/California/07/2009. Meanwhile, a Glu-to-Gly substitution in the NP protein also occurred in mut-A/California/07/2009 virus. Besides these two mutations occurred between the chicken embryonated eggs, which were derived from viruses with corresponding wild type viruses, two variations between the two wild type viruses also have been noticed. Compared with A/California/04/2009, A/California/07/2009 possessed an Ala instead of Thr at position 197 of the HA protein, and possessed a Tyr instead of Phe at position 351 of the NA protein. These two variations also existed between mut-A/California/04/2009 and mut-A/California/07/2009 viruses.

**Table 1:** Genomic sequences alignment of the wild type and mutated A/California/04/2009 and A/California/07/2009 viruses.

Viruses	PB2	PB1	PA	HA				NP		NA		M2&M1	NEP&NS1
	
				nt	aa	nt	aa	nt	aa	nt	aa		
	
				640	197	716	222	341	114	1052	351		
A/California/04/2009	-	-	-	A	Thr	A	Asp	A	Glu	T	Phe	-	-

mut-A/California/04/2009	-	-	-	A	Thr	**G**	**Gly**	A	Glu	T	Phe	-	-

A/California/07/2009	-	-	-	G	Ala	A	Asp	A	Glu	A	Tyr	-	-

mut-A/California/07/2009	-	-	-	G	Ala	**G**	**Gly**	**G**	**Gly**	A	Tyr	**-**	-

### In vitro comparison of virus replication in cells

**Figure 1:**
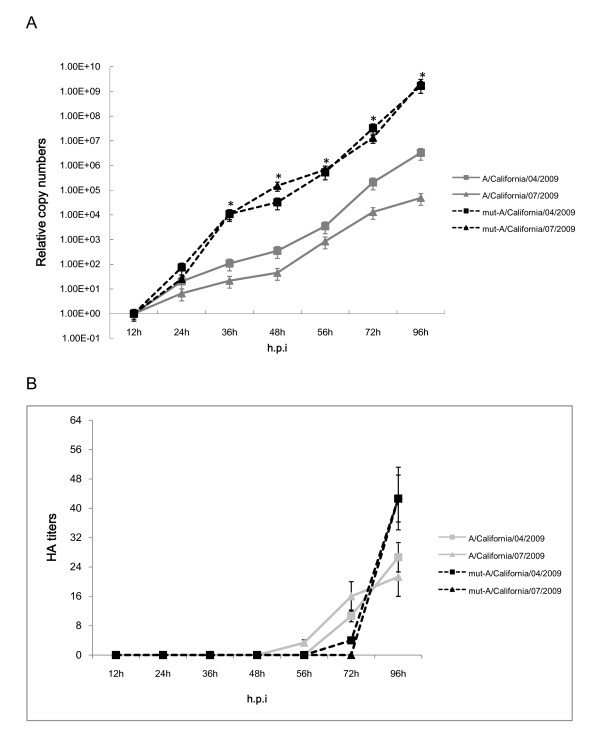
**Replication kinetics of the wild type and mutated A/California/04/2009 and A/California/07/2009 viruses *in vitro***. (A) Viral RNA loads of viruses cultured in MDCK cells. Data represented the percentage of mean viral RNA load per 100 μl of infected cell supernatant ± standard deviation. (B) Hemagglutination assays of viruses cultured in MDCK cells. Data represented the mean viral HA titers per 100 μl of infected cell supernatant ± standard deviation. * represent statistical significance at *P *< 0.05 (one-way ANOVA).

Statistical analysis of the virus replication kinetics in MDCK cells by one-way ANOVA showed that mut-A/California/04/2009 and mut-A/California/07/2009 viruses exhibited significant higher (*P *< 0.05) viral RNA load in infected cells than wild type A/California/04/2009 and A/California/07/2009 from 36 to 96 h.p.i, with peak load of roughly 3-4 log higher than respective wild type viruses. However, there were no statistical differences (*P *> 0.05) among viral RNA load of A/California/04/2009 with A/California/07/2009, and mut-A/California/04/2009 with mut-A/California/07/2009 viruses at each time point (Figure 1A). Furthermore, the results of hemagglutination assay in MDCK cells showed that mut-A/California/04/2009 and mut-A/California/07/2009 viruses also exhibited higher ability to agglutinate turkey erythrocyte cells than the wild type viruses at 96 h.p.i (*P *< 0.05), whereas there were no statistical differences (*P *> 0.05) among the hemagglutination results at 72 h.p.i. of all viruses. All viruses were unable to agglutinate turkey erythrocyte cells before 48 h.p.i (Figure 1B).

### In vivo comparison of virulence in the mice model

**Figure 2:**
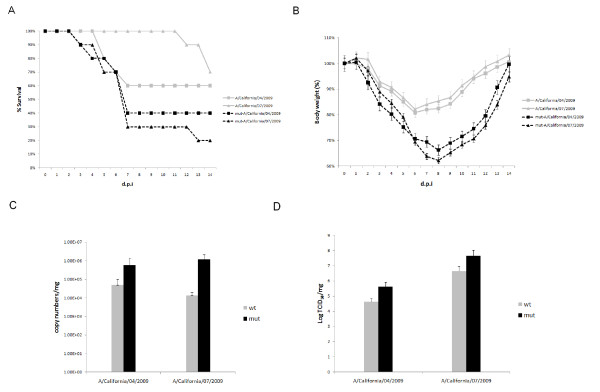
**Virulence comparison of the wild type and mutated A/California/04/2009 and A/California/07/2009 viruses in BALB/c mice**. (A) Survival percentage of mice intranasally challenged with each virus with 10^6 ^TCID_50 _in a volume of 50 μl. (B) Body weight changes of infected mice. Mean body weight and standard deviation were calculated as percentage of body weight compared to those at 0 d.p.i. (C) The viral RNA loads of viruses in lung tissues of mice which euthanized at 5 d.p.i.. Data represented the mean viral RNA load per microgram of lung tissues ± standard deviation. (D) The viral titers in lung tissues of mice which euthanized at 5 d.p.i.. Data represented the mean log TCID_50 _per microgram of lung tissues.

To further compare the virulence of the wild type and mutant viruses *in vivo*, we challenged BALB/c mice with each virus intranasally to evaluate mortality, weight loss, viral titer and RNA load in the lung tissue. The percentages of mice surviving the infection were showed in Figure 2A. 80% of mice which were inoculated with mut-A/California/07/2009 virus died until 14 d.p.i.. Mice infected with mut-A/California/04/2009 showed a survival ratio of 40%, whereas the lethality rates of A/California/04/2009 and A/California/07/2009 viruses were reduced to 40% and 30% respectively. Meanwhile, mut-A/California/04/2009 and mut-A/California/07/2009 viruses caused nearly 40% body weight loss by 9 d.p.i. when the mice started to regain the weight over the course of the remaining observation period. However, the body weight losses of mice which were challenged with wild type A/California/04/2009 and A/California/07/2009 were less than 20% (Figure 2B). To further investigate the differences in pathogenesis of all viruses in mice, we examined the viral RNA loads in lung tissues of challenged mice. The mean viral RNA load (copy numbers per mg ± SD) (Figure 2C) and viral titers (TCID_50 _per mg ± SD) (Figure 2D) were determined on 5 d.p.i. when virus shedding reached its peak. Mice inoculated with mut-A/California/04/2009 and mut-A/California/07/2009 viruses resulted in 1-2 log higher RNA copy numbers of virus in the lungs than respective wild type viruses. Consistent with the viral RNA load results, lung tissues of mice which challenged with mut-A/California/04/2009 and mut-A/California/07/2009 exhibited 2 log higher viral titers compared with those inoculated with the wild type viruses.

**Figure 3:**
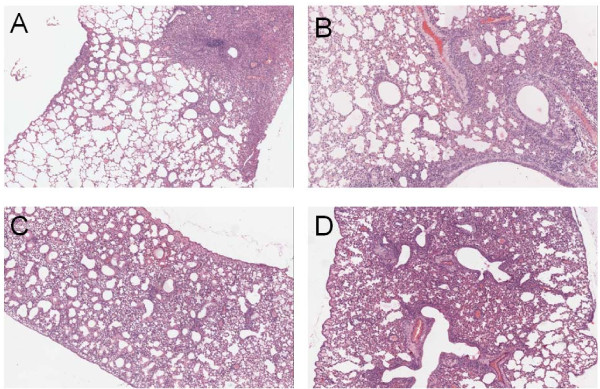
**Pathological analysis of the lung tissues of challenged BALB/c mice**. The images were obtained on an OLYMPUS BX-50 light microscope with 40× magnification. (A) Lung tissues of mice challenged with A/California/04/2009 virus; (B) Lung tissues of mice challenged with A/California/07/2009 virus; (C) Lung tissues of mice challenged with mut-A/California/04/2009 virus; (D) Lung tissues of mice challenged with mut-A/California/07/2009 virus.

Furthermore, to examine the pathological changes in the lungs of challenged mice, lungs were isolated on 5 d.p.i., and serial sectioned and stained with hematoxylin and eosin (H&E). Pathological examination revealed all viruses replicated efficiently in the lung tissues of mice, which all exhibited inflammatory hyperaemia, hemorrhage, edema, and exudative pathological changes. Pathological changes appeared in more than 85% lung tissues of mice inoculated with mut-A/California/04/2009 and mut-A/California/07/2009 viruses, whereas for those mice inoculated by A/California/04/2009 and A/California/07/2009 viruses, the lesion occurrence rate in lung tissues is ranged from 60% to 85% (Figure 3).

## Discussion

The 2009 pandemic A (H1N1) virus is generally associated with mild disease and a relatively low mortality. However, sporadic severe or fatal cases were reported from time to time. Apart from individual immunity discrepancy, it is projected that the high virulence and pathogenesis of influenza strains also is caused by the specific sequence of viral proteins and mutations occurred in critical regions [8,9], including the external surface glycoproteins, hemagglutinin (HA) and neuraminidase (NA) in relation to their interaction with sialic acids, the viral specific receptor on host cells [3,10-12]. Meanwhile, the three polymerase proteins have also been shown to be important virulence determinants of H5N1 and H7N7 viruses and transmission of the 1918 H1N1 virus [10,11,13,14]. Furthermore, the two nonstructural proteins PB1-F2 [15,16] and NS1 [17] are also implicated in virulence of H5N1 and 1918 H1N1 viruses. For the 2009 pandemic A (H1N1) virus, it is also supposed that mutations in specific regions of the viral genome could result in more virulent viruses, leading to a more severe pandemic.

In this study, two prototypic strains of the 2009 pandemic A (H1N1) influenza viruses, A/California/04/2009 and A/California/07/2009, were continuous passaged in chicken embryonated eggs, and the replication kinetics and pathogenecity between the embryonated chicken eggs adaptive viruses and the corresponding wild type viruses were compared *in vitro *and *in vivo*. We first examined the viral replication kinetics in MDCK cells and found enhanced replication of the mut-A/California/04/2009 and mut-A/California/07/2009 viruses, which leading to increased peak RNA load of toughly 3-4 log than their wild type viruses. Meanwhile, hemagglutinating assay showed that the peak viral titers in MDCK cells were similar with the situation of viral RNA load results. On the other hand, our *in vivo *experiment was conducted in BALB/c mice model. Pronounced difference in virulence was observed. Similarly, mut-A/California/04/2009 and mut-A/California/07/2009 exhibited significant enhanced virulence as compared to the wild type strains because of higher mortality, body weight loss, viral RNA copy numbers and titers, and more severe pathological changes in mice lungs tissues.

Sequence alignment results showed that there was only one single mutation in the HA protein (Asp222Gly) between wt- and mut- A/California/04/2009 viruses, and two mutations respectively in the HA (Asp222Gly) and NP (Glu114Gly) protein between wt- and mut- A/California/07/2009 viruses. It is interesting to note that the two embryonated eggs derived adaptive viruses mutated at the same site and substituted by the same amino acid in the HA protein. According to previous report, the egg adaptation process frequently introduces amino acid changes into the HA protein that affect viral receptor binding and sometimes even viral antigenicity [18-22]. Meanwhile, residue 222 was reported to locate in the receptor binding domain of the HA protein [23-26]. As for other HA protein structures, the receptor binding domain is composed of three structural elements: a 190-helix (residues 184-191), a 220-loop (residues 218-225), and a 130-loop (residues 131-135) [27]. We proposed that the substitution of Asp with Gly at residue 222 of the HA protein may favor the interaction with the specific receptor sialic acid α2,6Gal and further virion entry into the host cells. Indeed, this hypothesis has been verified by some studies. The HA from the 1918 H1N1 pandemic switched from avian to human receptor specificity through mutation at two positions (Glu187Asp and Gly222Asp) [4]. In addition, the A/New York/1/18 strain of the 1918 pandemic possessed a Gly at position 222 and this markedly affected receptor binding, reduced α2,6 preference and increased α2,3 avidity [4]. More recently, there has been focus on the possible role of the mutation at position 222 of the HA protein and its role in severe clinical outcome [28,29]. The Asp222Gly and Asp222Asn single and mixed variants were found in pandemic viruses as well as direct sequencing from clinical specimens collected throughout the 2009 pandemic from approximately 20 countries, including Norway, Mexico, Ukraine and the USA. Summarized the above data, it is much likely that Asp222Gly substitution in the HA protein may be a critical positive selection event during the virus egg amplification process and is an important virulence factor for the 2009 pandemic A (H1N1) influenza virus. Polymorphism at this residue may have remarkable impact on viral host range, replication, and pathogenicity. It is worth noting that although the Asp222Gly mutation currently has not been associated with severe pandemic in humans, our data suggests that such laboratory adaptation of the pandemic A (H1N1) viruses from human to chicken embryonated eggs may increase the potential risks involved in handling with the mutant viruses. Once they cross species and transmit from chicken back to human, there may be another potential severe pandemic of influenza.

## Conclusions

In summary, we found that when the 2009 pandemic A (H1N1) influenza virus were propagated in the chicken embryonated eggs, a substitution of Asp-to-Gly at position 222 in the HA protein was prone to occur under positive selection pressures, and this single amino acid mutation could dramatically increase the virus replication ability *in vitro *and pathogenicity *in vivo*. These preliminary observations, however, invite further studies into the transmission and evolution of these viruses, and provide new insights into the impact of genetic changes that may have on the replication and pathogenicity of the 2009 pandemic A (H1N1) influenza virus.

## Competing interests

The authors declare that they have no competing interests.

## Authors' contributions

LLX carried out the genome sequencing and analysis, real-time PCR, statistical analysis and drafted the manuscript. QL performed the cell infection and hemagglutination assay. WD, FDL, and YLM participated in the animal challenge. HZ and CMM performed pathological analysis. LJZ carried out follow-up. CQ and LLB predicated in the design of the study. All authors read and approved the final manuscript.
